# A Rare Case of Coexistence: Discoid Lupus Erythematosus and Sturge–Weber Syndrome

**DOI:** 10.1002/ccr3.71633

**Published:** 2025-12-09

**Authors:** Amir Efazati, Yousof Mir, Shiva Pouradeli, Zahra Abdolahinia, Mahdi Sharifzadeh Kermani

**Affiliations:** ^1^ Clinical Research Development Unit, Shafa Hospital Kerman University of Medical Sciences Kerman Iran; ^2^ Kerman Neuroscience Research Centre, Institute of Neuropharmacology Kerman University of Medical Sciences Kerman Iran; ^3^ Neurosciences Research Center Isfahan University of Medical Sciences Isfahan Iran; ^4^ Student Research Committee Kerman University of Medical Sciences Kerman Iran

**Keywords:** comorbidity, diagnosis, discoid lupus erythematosus, inflammation mediators, Sturge–weber syndrome

## Abstract

This rare coexistence of Sturge–Weber Syndrome (SWS) and discoid lupus erythematosus (DLE) presents unique diagnostic and therapeutic challenges. It highlights potential shared environmental triggers and overlapping inflammatory mechanisms, underscoring the need for a multidisciplinary approach to management. Further research is essential to clarify their interaction and improve patient outcomes.

## 1 Introduction

Lupus erythematosus is a complex autoimmune disease that affects multiple organ systems, primarily manifesting through cutaneous involvement. Discoid lupus erythematosus (DLE), the most common chronic cutaneous subtype, is characterized by well‐demarcated, erythematous, scaly plaques that may lead to scarring, pigmentary changes, and permanent alopecia, particularly on sun‐exposed areas such as the face, scalp, and ears. Although systemic involvement is less frequent in DLE compared to systemic lupus erythematosus (SLE), approximately 20% of patients may eventually develop systemic manifestations [1, 2, 3, 4].

Sturge–Weber syndrome (SWS), is a rare congenital neurocutaneous disorder caused by somatic mosaicism, typically presenting with neurological or ocular manifestations, including seizures, migraines, glaucoma, and buphthalmos [5]. The coexistence of DLE and SWS is exceedingly rare, with only a few documented cases in the literature [6]. This uncommon association poses diagnostic and therapeutic challenges, as the vascular abnormalities of SWS may influence local immune responses or skin barrier function, potentially exacerbating autoimmune skin disorders such as DLE. Moreover, SWS may complicate the clinical course of lupus due to increased neuropsychiatric and thromboembolic risks [7]. Here, we present a noteworthy case illustrating the coexistence of these two conditions. To our knowledge, similar cases have only been reported by Nikyar et al. and Jethwa et al. [3, 7].

### 1.1 Case History and Examination

**FIGURE 1:**
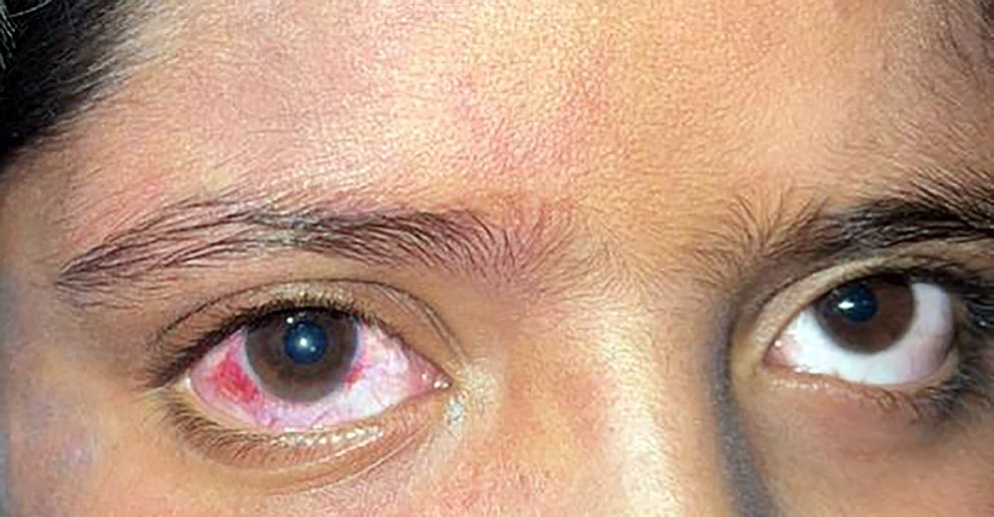
Cutaneous manifestations of Sturge–Weber syndrome and discoid lupus erythematosus. Port‐wine stains involving the right facial dermatomes, with erythematous and scaly discoid lesions in the preauricular region.

**FIGURE 2:**
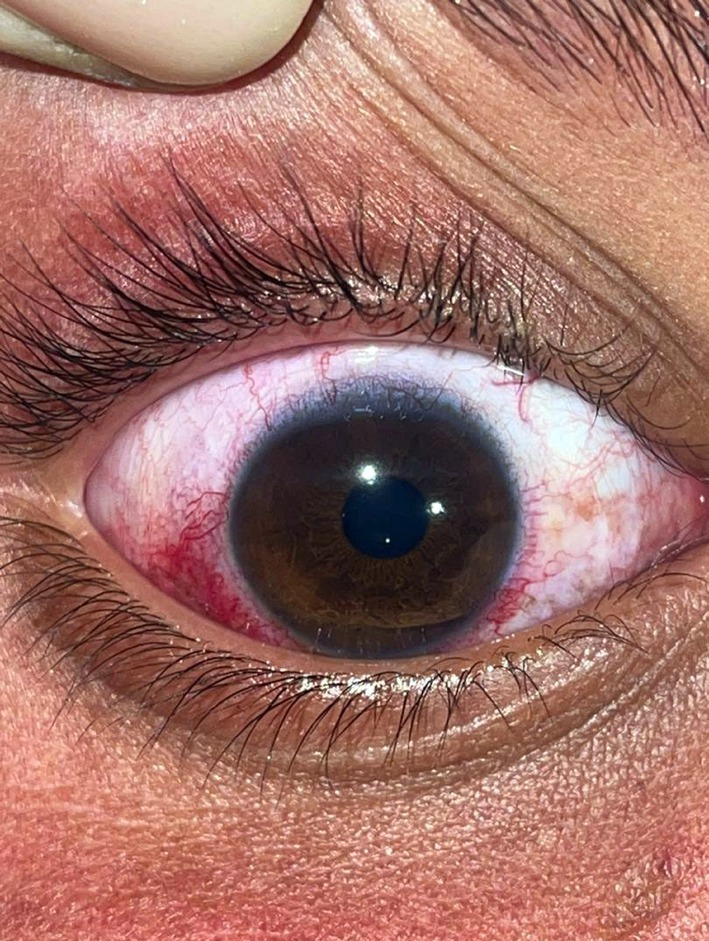
Conjunctival vascular changes. Bulbar conjunctiva showing diffuse vascular congestion and erythema, consistent with conjunctival injection.

An eleven‐year‐old boy with a known diagnosis of SWS presented to the ophthalmology clinic. He exhibited no neurological abnormalities; however, a port‐wine stain was observed on the right side of his face, involving the V1, V2, and V3 dermatomes. Multiple discoid patches, hyperpigmented and atrophic in nature, were noted in the preauricular region, with erythematous lesions predominantly affecting the right side. Erythematous lesions were present in various locations, predominantly on the right side. Both sclerae appeared pink, more prominently on the right, consistent with vascular congestion associated with SWS (Figures 1 and 2).

## 2 Investigations, Differential Diagnosis, and Treatment

### 2.1 Cardiology and Neurology Assessments

Comprehensive cardiology and neurology evaluations revealed no significant abnormalities. Although neuroimaging (MRI/CT) data were unavailable due to incomplete records, the diagnosis of Sturge–Weber syndrome was supported by the characteristic facial vascular distribution and ophthalmologic findings.

### 2.2 Ophthalmology Assessments

**FIGURE 3:**
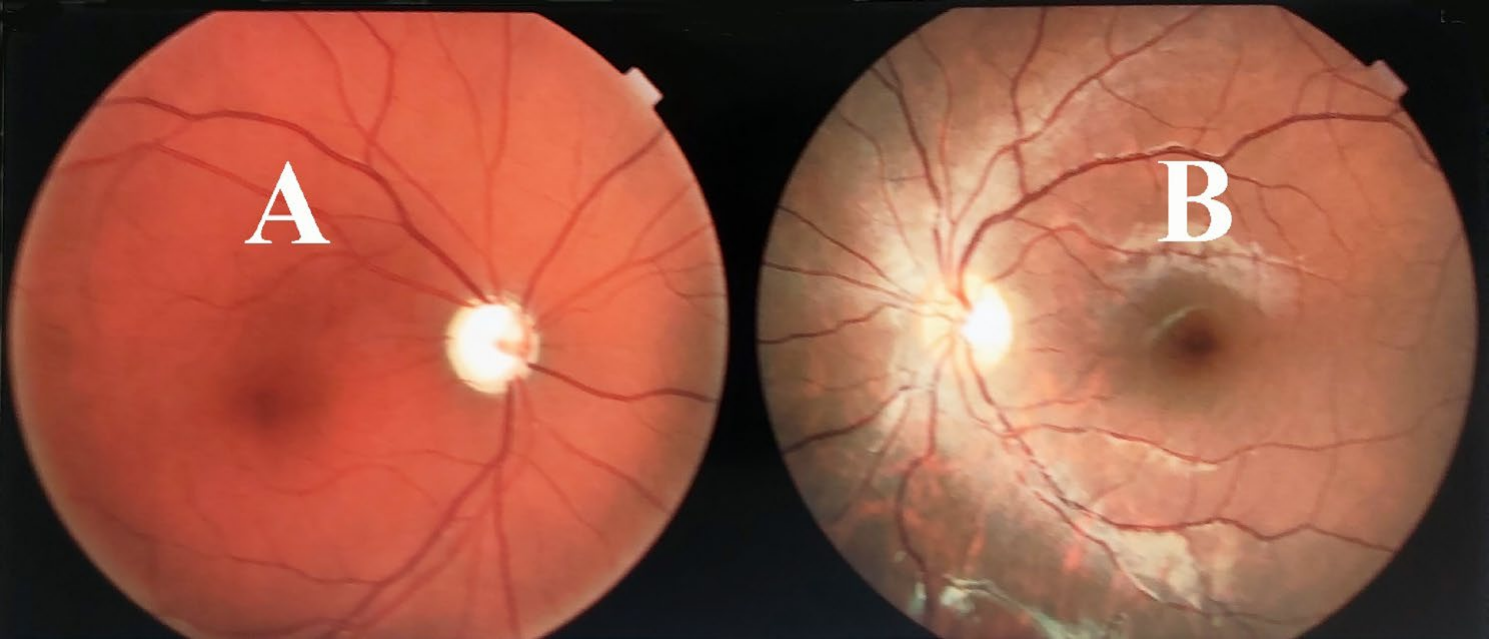
The right eye (A) shows total cup optic disc, consistent with diffuse retinal nerve fiber layer (RNFL) thinning confirmed by OCT. The left eye (B) appears within normal.

A detailed ophthalmologic examination was performed, including indirect ophthalmoscopy and visual acuity testing using a Snellen chart (Figure 3).

**FIGURE 4:**
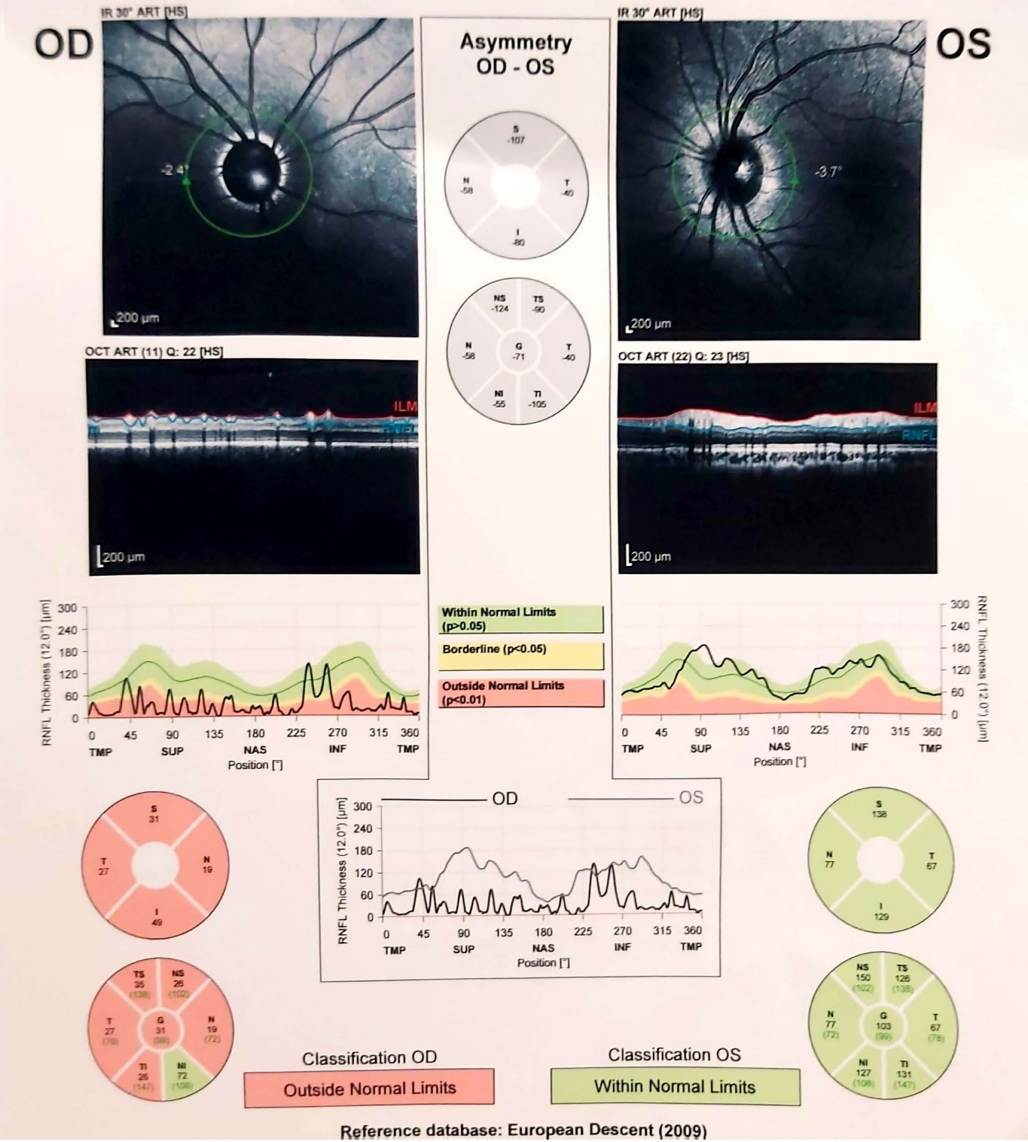
Peripapillary optical coherence tomography (OCT) analysis of both eyes. Optical coherence tomography of the retinal nerve fiber layer (OCT RNFL) in the right (OD) and left (OS) eyes. The right eye shows diffuse RNFL thinning, most pronounced in the superior and temporal quadrants, while the left eye demonstrates RNFL thickness within normal limits.

Optical coherence tomography (OCT) was subsequently conducted to evaluate the macular and peripapillary retinal nerve fiber layers and any potential changes affecting visual quality (Figure 4).

**FIGURE 5:**
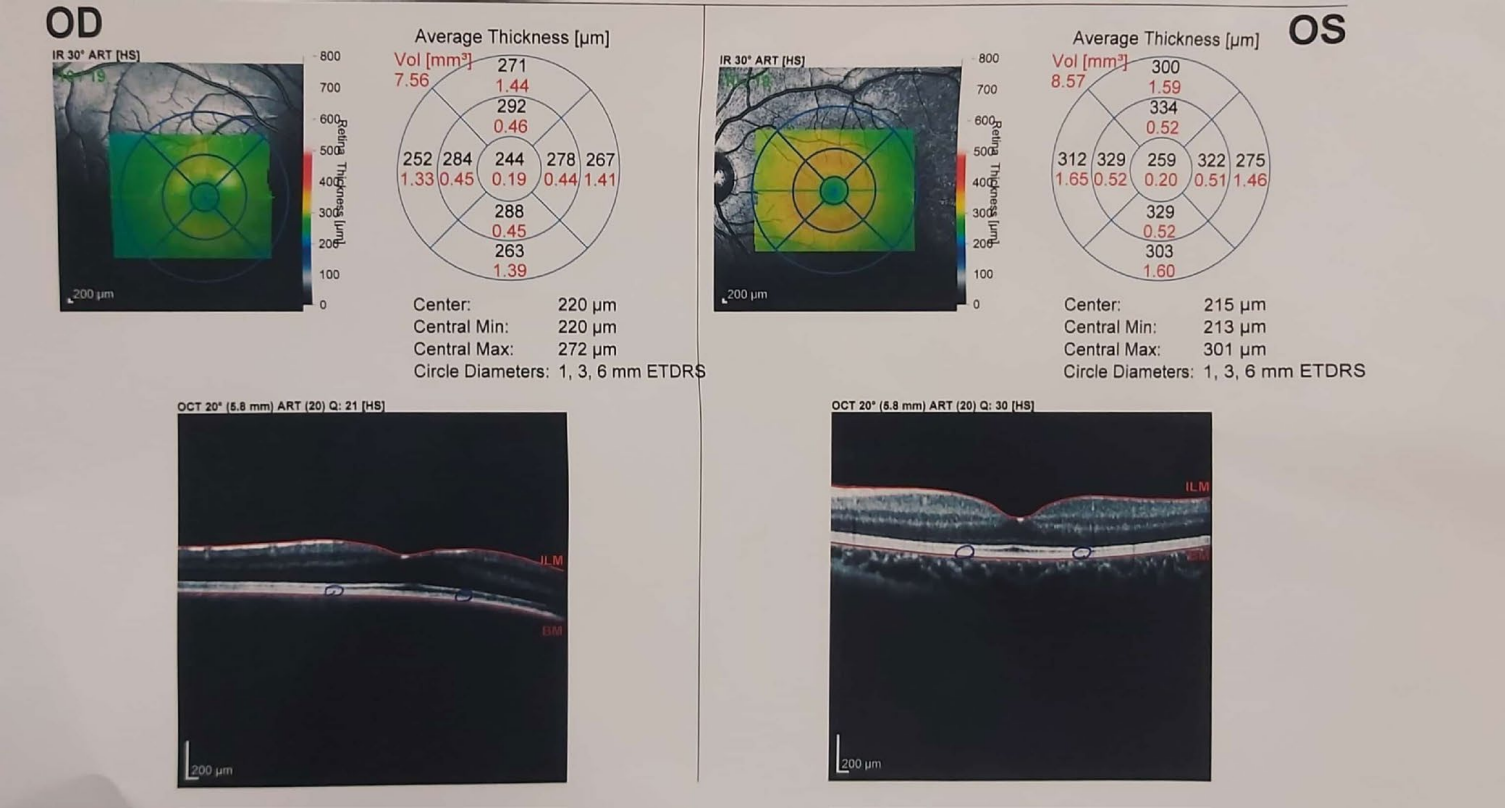
Macular optical coherence tomography of the right (OD) and left (OS) eyes. The right eye shows mild diffuse macular thinning especially in ganglion cell layer (GCL) and inner plexiform layer (IPL), while the left eye demonstrates values within normal limits.

The peripapillary OCT revealed decreased retinal nerve fiber layer thickness in the right eye across all quadrants. Although the nasal‐inferior region showed reduced thickness, it remained within the limit of normal. The left eye demonstrated normal thickness in all regions. Macular OCT showed a mild reduction in thickness without clinically significant visual impairment (Figure 5).

### 2.3 Pathology Assessments

**FIGURE 6:**
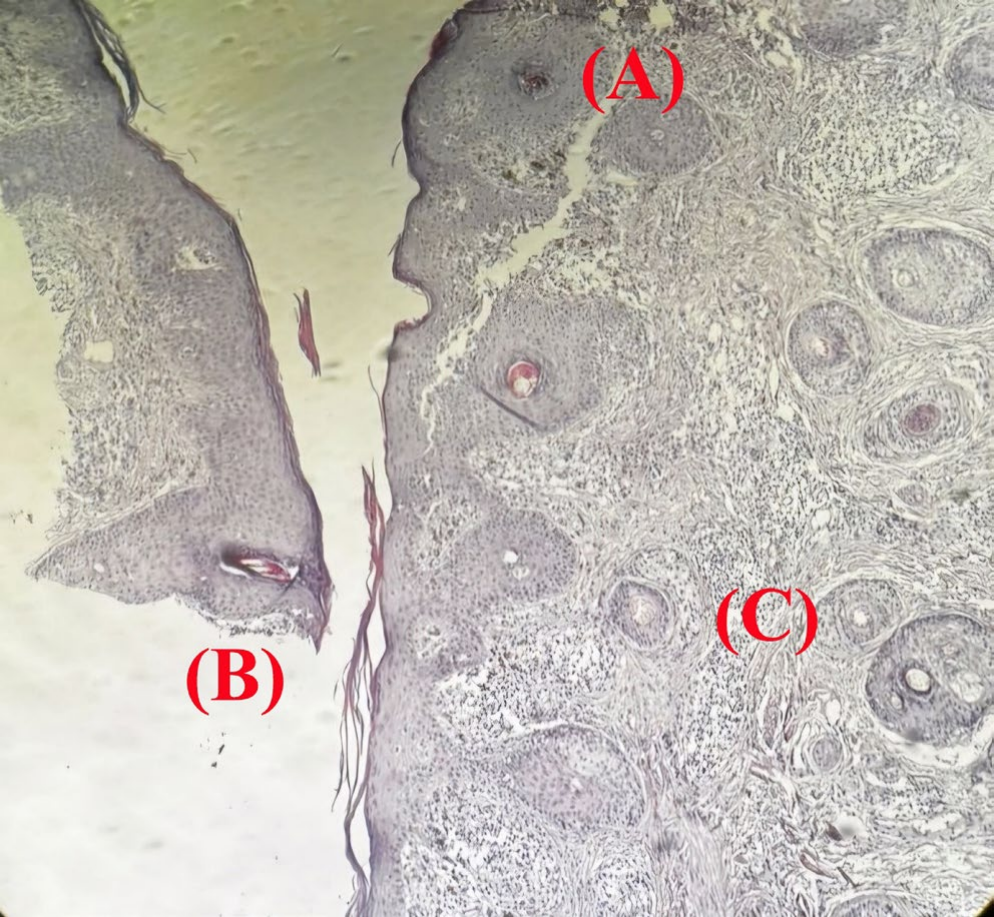
Histopathology of discoid lupus erythematosus. Hematoxylin and eosin–stained section showing (A) hyperkeratosis with follicular plugging, (B) hydropic degeneration of the basal layer, and (C) dense perivascular lymphocytic infiltration—features characteristic of discoid lupus erythematosus.

**FIGURE 7:**
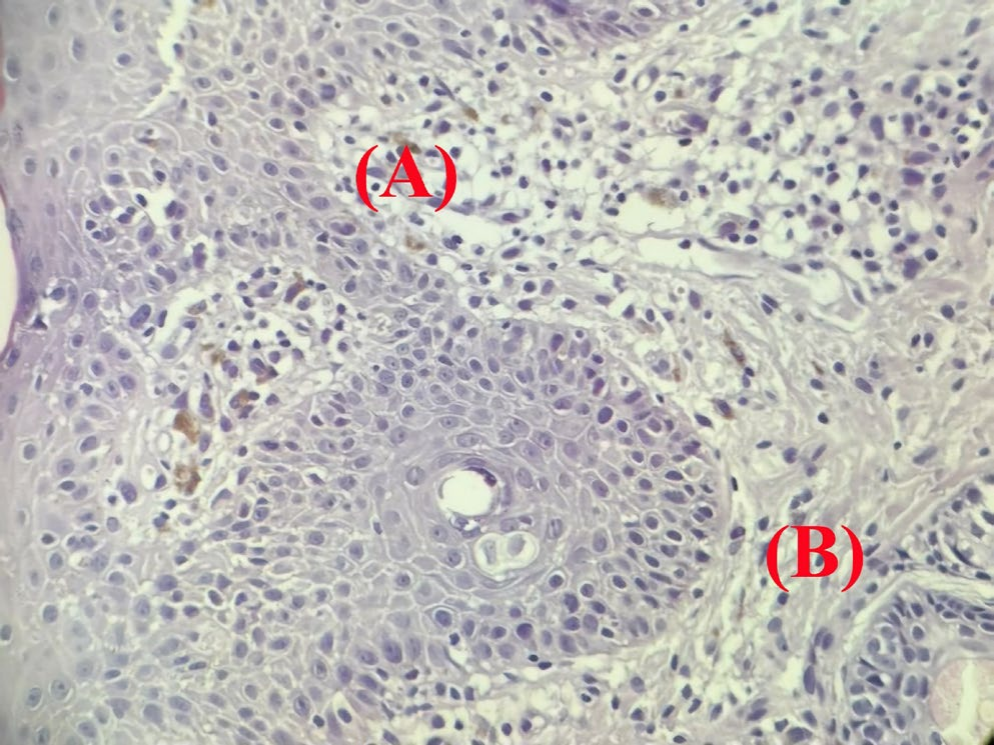
High‐power histopathology of interface dermatitis. Hematoxylin and eosin–stained section showing (A) hydropic degeneration of the basal layer and (B) melanin incontinence in the superficial dermis—findings consistent with discoid lupus erythematosus.

Histopathological analysis demonstrated hyperkeratosis, follicular plugging, hydropic degeneration of the basal layer, and focal epidermal thinning. The dermis exhibited melanin incontinence, solar elastosis, and perivascular and periappendageal inflammatory infiltrates (Figures 6 and 7).

While the port‐wine stains and scleral discoloration were consistent with SWS. a 4 mm punch biopsy from the preauricular and buccal discoid patches was performed to confirm the diagnoses. Differential diagnoses included:

1. Discoid lupus erythematosus
2. Lichen planus
3. Keratosis lichenoides chronica

## 3 Results, Outcome, and Follow‐Up

The patient remained seizure‐free and exhibited no neurological or cardiac abnormalities. He was referred to a dermatologist for ongoing management of discoid lupus erythematosus and scheduled for regular ophthalmologic follow‐up. A multidisciplinary care plan ensured comprehensive monitoring and highlighted the importance of vigilance for potential complications associated with SWS.

## 4 Discussion

In this report, we describe a rare coexistence of SWS and DLE. SWS is a congenital neurocutaneous disorder typically diagnosed at birth, whereas DLE represents the most common form of chronic cutaneous lupus erythematosus. To our knowledge, this is the first documented case addressing the simultaneous occurrence of these two distinct conditions.

Nikyar et al. reported a 3‐month‐old girl with both SWS and neonatal lupus erythematosus (NLE). The patient exhibited facial port‐wine stains without cardiac abnormalities, and histopathology revealed a collagenous stroma infiltrated by spindle and mast cells. Interestingly, the findings diverged from typical presentations, showing histological overlap with conditions such as progressive mucinous histiocytosis, self‐healing juvenile cutaneous mucinosis, and cutaneous mucinosis of infancy [3].

Similarly, Jethwa et al. described an 11‐year‐old boy with SWS and childhood‐onset systemic lupus erythematosus (cSLE), who developed macrophage activation syndrome (MAS)—a life‐threatening hyperinflammatory state characterized by persistent high‐grade fever, hemorrhagic manifestations, and lymphadenopathy [7].

In our patient, discoid lupus lesions were localized in the preauricular area and demonstrated keratinization and hyperpigmentation, while the mucosal surfaces remained unaffected. Although Admani et al. noted that ultraviolet (UV) radiation can exacerbate DLE lesions [8], our patient, lived in a low‐income rural area with significant outdoor exposure and inadequate photoprotection, likely contributing to disease exacerbation.

SWS is frequently associated with ocular manifestations, particularly glaucoma, which may develop early or later in life [9]. However, our patient showed no visual impairments. Nevertheless, given the potential for glaucoma and reduced retinal nerve fiber layer thickness, continuous ophthalmologic follow‐up remains essential.

### 4.1 Potential Pathophysiological Interactions

Although direct studies exploring the interaction between SWS and DLE are lacking, several mechanisms may explain their coexistence.

### 4.2 Mechanisms of DLE

1. *Autoimmune response*: DLE results from an autoimmune attack on keratinocytes, leading to inflammation and scarring. Environmental factors—especially UV exposure—commonly trigger lesions exacerbation and play a central role in disease pathogenesis [10].
2. *Genetic susceptibility*: Genetic predisposition contributes to DLE through variants that impair immune regulation and apoptotic cell clearance [11].
3. *Cytokine dysregulation*: Overexpression of type I interferons and other pro‐inflammatory cytokines drives the chronic inflammation characteristic of DLE [12].

### 4.3 Mechanisms of SWS

1. *Vascular malformation*: SWS arises from a postzygotic *GNAQ* gene mutation, causing abnormal capillary–venous development in the skin (port‐wine stains), brain (leptomeningeal angiomas), and eyes [13].
2. *Neurological sequelae*: These vascular malformations can result in seizures and developmental delays due to chronic hypoperfusion and neural injury [14].
3. *Inflammatory pathways*: Inflammatory processes involving vascular and neural tissues may overlap with autoimmune mechanisms, potentially linking SWS to autoimmune phenomena such as lupus [15].

### 4.4 Possible Interactions

1. *Shared environmental triggers*: Both conditions may be exacerbated by environmental factors—most notably ultraviolet (UV) radiation—which is a well‐known trigger of DLE lesions and may also modulate the vascular and inflammatory processes implicated in SWS.
2. *Autoimmunity and inflammation*: The inflammatory milieu in SWS may interact with the autoimmune mechanisms underlying DLE. Direct evidence for their co‐occurrence is limited; however, systemic lupus erythematosus (SLE) has been reported in some patients with SWS, suggesting a potential overlap in underlying pathophysiology [7].

In summary, although DLE and SWS have distinct pathophysiological bases, their coexistence may be promoted by shared environmental exposures and overlapping inflammatory pathways. Further mechanistic and epidemiological studies are required to determine whether this association reflects shared triggers, direct pathophysiological interactions, or chance comorbidity.

### 4.5 Complications of Coexisting DLE and SWS

The coexistence of DLE and SWS may predispose patients—particularly children—to several complications:

1. *Thromboembolic risk*: Both independently increase thromboembolic tendencies, and their coexistence may amplify this risk, particularly in lupus patients with pro‐inflammatory and pro‐coagulant states [7].
2. *Neuropsychiatric manifestations*: Patients with both DLE and SWS may experience neuropsychiatric issues. The neurological features of SWS can be aggravated by lupus‐associated neuroinflammation, resulting in seizures, cognitive deficits, or mood disturbances [7].
3. *Macrophage activation syndrome (MAS)*: A severe complication in cSLE that may be precipitated by SWS‐related immune dysregulation, MAS can lead to multi‐organ failure and requires prompt recognition [7].
4. *Diagnostic complexity*: Overlapping clinical and histopathological features make it difficult to distinguish between manifestations of DLE, SWS, or both, delaying appropriate diagnosis and management [3].

### 4.6 Importance of Early Diagnosis

Early diagnosis of DLE is crucial to prevent progression to systemic lupus erythematosus (SLE) and minimize scarring and morbidity [16]. Likewise, timely identification of SWS is vital for the management of seizures and cognitive complications [17].

Early recognition of both disorders enables individualized treatment—photoprotection and immunosuppressive therapies for DLE, and antiepileptic or ocular interventions for SWS [7, 15].

A multidisciplinary approach involving dermatologists, neurologists, and ophthalmologists ensures comprehensive care. Continuous follow‐up, patient education, and regular screening for overlapping complications are essential to improving quality of life and long‐term outcomes [7, 15, 17, 18].

## 5 Conclusion

This report presents a rare coexistence of SWS and DLE, emphasizing the diagnostic and therapeutic challenges inherent in managing both conditions concurrently. While SWS arises from vascular malformations linked to GNAQ mutations, and DLE represents an autoimmune process triggered by environmental factors such as UV exposure; shared inflammatory and vascular mechanisms may underlie their intersection.

This overlap can increase the risk of thromboembolic, neuropsychiatric, and immunologic complications, particularly in pediatric patients. A multidisciplinary management strategy and vigilant long‐term monitoring are essential for optimal outcomes. Further research is warranted to elucidate the mechanisms of this rare coexistence and to develop evidence‐based management guidelines.

## 6 Limitations

This case report has several limitations. Firstly, the lack of longitudinal follow‐up data limits our ability to assess the long‐term disease progression and treatment outcomes. The patient was lost to follow‐up after the initial evaluation, restricting insight into potential complications.

Second, while OCT provided valuable structural information, the absence of additional imaging—such as fundus photography or visual field testing—limited a comprehensive assessment of ocular involvement. Reliance on clinical and histopathological findings without imaging corroboration may reduce diagnostic certainty.

Finally, due to the rarity of SWS coexisting with DLE, broad generalizations cannot be made. Nonetheless, this case adds to the limited body of literature documenting this unique overlap, highlighting the importance of meticulous clinical evaluation and multidisciplinary management in similar presentations.

## Author Contributions

**Amir Efazati:** data curation, investigation, validation, writing – original draft. **Yousof Mir:** investigation, methodology, writing – original draft. **Shiva Pouradeli:** investigation, writing – review and editing. **Zahra Abdolahinia:** writing – review and editing. **Mahdi Sharifzadeh Kermani:** data curation, project administration, supervision, validation, writing – review and editing.

## Funding

The authors have nothing to report.

## Ethics Statement

This study was conducted in accordance with the ethical principles outlined in the Declaration of Helsinki and received approval from the Institutional Review Board (IRB) of Kerman University of Medical Sciences. Prior to inclusion in the study, the guardians of the patient were thoroughly informed about the study's objectives, procedures, potential risks, and anticipated benefits. Written informed consent was obtained from the guardians after confirming that they fully understood the nature of the study and voluntarily agreed to allow their child to participate. The consent process emphasized the child's autonomy, privacy, and confidentiality, adhering to the highest ethical standards for research involving human participants.

## Consent

Written informed consent was obtained from the guardians for the patient's participation in this study. The consent form included detailed information about the study's purpose, procedures, potential risks, benefits, and the voluntary nature of participation. The guardians of the patient provided written consent for the publication of this case report and any accompanying images. This consent ensures that they understand and agree that the data will be published in a medical journal and made accessible to the scientific community.

## Conflicts of Interest

The authors declare no conflicts of interest.

## Data Availability

The data supporting the findings of this case report are available from the corresponding author upon reasonable request. All relevant clinical information and patient details have been anonymized to protect patient confidentiality.
